# Regular moderate physical exercise decreases Glycan Age index of biological age and reduces inflammatory potential of Immunoglobulin G

**DOI:** 10.1007/s10719-023-10144-5

**Published:** 2023-12-26

**Authors:** Nina Šimunić-Briški, Vedran Dukarić, Mateja Očić, Tomislav Madžar, Martina Vinicki, Azra Frkatović-Hodžić, Damir Knjaz, Gordan Lauc

**Affiliations:** 1 Genos Ltd, 10000 Zagreb, Croatia; 2 Faculty of Kinesiology, University of Zagreb, 10000 Zagreb, Croatia; 3 Faculty of Pharmacy and Biochemistry, University of Zagreb, 10000 Zagreb, Croatia; 4 Vaš Pregled Sports and Occupation Medicine Polyclinic, 10000 Zagreb, Croatia; 5 University of Applied Health Sciences, 10000 Zagreb, Croatia

**Keywords:** Glycosylation, GlycanAge index, Biological age, Physical activity, Aging biomarkers

## Abstract

Physical inactivity and obesity are growing concerns, negatively impacting the general population. Moderate physical activity is known to have a beneficial anti-inflammatory effect. N-glycosylation of immunoglobulin G (IgG) reflects changes in the inflammatory potential of IgG. In this study, GlycanAge index of biological age (GlycanAge), one of the first commercially used biomarkers of aging, was employed to assess effects of exercise intensity in three different groups of athletes: professional competing athletes, regularly moderate active individuals and newly involved recreational individuals, compared to the group of inactive individuals. GlycanAge was significantly lower in the active group compared to the inactive group (β = -7.437, p.adj = 7.85E-03), and nominally significant and increased in professional athletes compared to the active group (β = 7.546, *p* = 3.20E-02). Competing female athletes had significantly higher GlycanAge comparing to active females exercising moderately (β = 20.206, p.adj = 2.71E-02), while the latter had significantly lower GlycanAge when compared with the inactive counterparts (β = -9.762, p.adj = 4.68E-02). Regular, life-long moderate exercise has an anti-inflammatory effect in both female and male population, demonstrated by lower GlycanAge index, and it has great potential to mitigate growing issues related to obesity and a sedentary lifestyle, which are relentlessly increasing world-wide.

**Supplementary Information:**

The online version contains supplementary material available at 10.1007/s10719-023-10144-5.

## Introduction

Physical inactivity and obesity are closely intertwined, representing two issues that heavily burden the health care systems in developed countries. Coupled with the increase in life expectancy, there is a growth in both the proportion and number of older adults in total world’s population. Currently, the population aged over 60 years is close to a billion, expected to double by 2050 [1].

Obesity is considered one of the modern age epidemics, advancing relentlessly and currently affecting over 2 billion worldwide [2]. Excessive nutrient intake, combined with insufficient physical activity, leads to a chronic positive caloric balance, resulting in increased adiposity that leads to obesity. This condition is directly linked to an increased risk of cardiovascular diseases (CVDs), type 2 diabetes (T2D) and other metabolic disorders. Moreover, it is linked with a reduction in aerobic capacity - cardiorespiratory fitness (CRF) [3]. The definition of CRF is described as the capacity of cardiovascular and respiratory systems to provide oxygen-enriched blood to working skeletal muscles and the muscles’ capacity to use oxygen for energy production to produce movement. CRF is used to determine cardiometabolic health, implying that interventions associated with increased physical activity, exercise training and the reduction of sedentary lifestyle would lead to its improvement. Physical inactivity is estimated to be the cause of 6–10% of premature mortality cases, related to CVD, T2D, breast and colon cancers. The global burden of physical inactivity is substantial, increasing rapidly, with the highest relative burden expected in the high-income countries [4].

Physical activity is a bodily movement produced by the contraction of the skeletal muscle that increases energy expenditure above the basal level, as defined by US Center for Disease Control and Prevention (CDC). Physical exercise triggers a cascade of inflammatory events, as every exercise bout leads to micro-damage in the working muscle. During and directly after exercise, a single bout of exercise will trigger an acute inflammatory response in the skeletal muscle, essential for muscle repair and regeneration. However, chronic systemic inflammation may affect and impair the acute anabolic exercise response [5].

The main hallmarks of aging skeletal muscle include the loss of strength and muscle mass, lessened regeneration, and weaker physical performance, along with insulin resistance, mitochondrial dysfunction, and impaired muscle metabolism. Sarcopenia, the decrease in muscle mass and strength, begins in the later stages of the third decade, continuing to the fourth decade of life. By the fifth decade, up to 10% of muscle mass can be lost due to atrophy, caused by a lack of physical activity. A significant decrease in muscle mass occurs after the fifth decade [6].

Aging is characterized by various processes, including muscle loss, a decrease in functional fitness, often followed by changes in body composition. As aging is associated with increased pro-inflammatory signaling, anti-inflammatory processes become impaired with aging. Macrophages within a skeletal muscle have an important role in repair and regeneration. The phenotypic transition of these macrophages from pro-inflammatory M1 to anti-inflammatory M2 is vital for attenuating the inflammatory response, leading to muscle regeneration, promoting myogenesis, and facilitating fiber fusion and growth. Older adults exhibit elevated basal levels of numerous pro-inflammatory cytokines, including IL-6. Immediately post exercise, older adults show greater levels of pro-inflammatory cytokine. In the 24–72 h post exercise period, macrophage infiltration is impaired, failing to recruit M1 macrophages. However, in the following 4–7 days, macrophage levels continuously increase [5]. Furthermore, inflammaging is an age-related process characterized by an increase in pro-inflammatory markers occurring in later stages of life. This is related to higher susceptibility to chronic morbidity, frailty, disability, and premature death. Two of the main treatment options, widely available yet underused, remain caloric restriction and physical activity [7]. Muscle contraction during exercise training induces the production and release of myokines (cytokines produced by muscle cells), including the proinflammatory interleukin 6 (IL-6), which can increase up to 100-fold after exercise [8]. Subsequently, IL-6 induces an increase in the production of the anti-inflammatory cytokines IL-1 receptor antagonist (IL-1ra) and IL-10, stimulating the occurrence of anti-inflammatory cytokines. IL-6 is involved in mediating the pro-inflammatory effects of both the innate and adaptive immune responses, attracting neutrophiles to the site of damage, while being involved in B and T-cell differentiation [9, 10]. Regular exercise training reduces basal IL-6 production, leading to decreased IL-6 plasma levels at rest and in response to exercise, considered to be a training adaptation [11]. Consequently, the introduction of aerobic training in a previously sedentary male population aged 60 and above resulted in significantly lower levels of circulating IL-6 after 3 months of exercise. Moreover, the levels of circulating Il-6 were even lower in age-matched lifelong male exercisers [12]. In professional athletes, cytokine production at severe intensities leads to changes in inflammatory parameters, including increased lactate, endotoxins and TNFα immediately after exercise, followed by higher concentrations of glucose and a trend for increased anti-inflammatory IL-10 one hour post exercise [8]. In moderate exercise, a marked increase in IL-6 and IL-10 occurs, leading to an anti-inflammatory effect by inhibiting TNFα and stimulating IL-1a. Additionally, muscle derived IL-6 produced by exercising muscles promotes improved glucose tolerance, ultimately having indirect long term anti-inflammatory effects through body composition improvement [9].

Immunoglobulin G (IgG), the most abundant antibody, has a central role in the adaptive immune system. IgG is secreted by plasma B cells and is found in all body fluids. It contains a fragment antigen-binding (Fab) and a fragment crystallizable (Fc) region, with the latter containing a conserved N-glycosylation site. Glycosylation is a ubiquitous posttranslational modification in which glycans are enzymatically added to various proteins and other molecules [13]. The addition of various IgG glycans acts as a switch between pro- and anti-inflammatory status of IgG. The IgG glycome accounts for all types of glycans present in one’s IgG molecules, and while it remains stable in homeostasis, it is very responsive to intrinsic and extrinsic environmental factors, aging, or disease development. In aging, the IgG glycome changes distinctively, which led to the development of Glycan Clock, the first biological age biomarker based solely on IgG glycans [14]. Following that discovery and further research into IgG glycosylation in health and disease, IgG glycans were proposed as novel biomarkers in personalized medicine. Many studies since revealed dynamic switches in the composition of IgG glycans, alternation from homeostasis to a proinflammatory in the early onset of various diseases. These conditions include cardiometabolic, autoimmune, neurological diseases; cancers, and infection related inflammation [13]. Additionally, changes related to sex hormones have been observed, with these hormones driving significant shifts during the regular menstrual cycle in women, as well as in menopause, where it was shown that menopause increases biological age, while hormone supplementation reduces it back to a premenopausal state [15]. Further-more, it has been demonstrated that a reduction in body fat in overweight population leads from pro-inflammatory to anti-inflammatory shift [16–18]. Exercise training has a similar anti-inflammatory effect, which is more pronounced in the young population. Tijardović et al. showed that intense physical exercise induces an anti-inflammatory shift in IgG glycans in young male population [19], while Sarin et al. demonstrated that intense physical exercise coupled with caloric restriction in normal BMI female population led to a pro-inflammatory shift, that returned to baseline values in the recovery period [20]. Even though exercise is considered to evoke anti-inflammatory effects, in the middle-aged, sedentary, overweight population, three months of moderate physical activity didn’t have the desired effects in that short period, as metabolic changes take longer [21]. In this cross-sectional study, we investigated the impact of exercise duration and intensity on GlycanAge index of biological age (GlycanAge), one of the first commercially used biomarkers of aging, used to track responses to lifestyle interventions [22].

## Materials and methods

### Participants

The study population included a total of 276 apparently healthy participants, divided into four different groups, depending on their physical activity level. In the inactive group (INACT) there were 99 participants (F = 42 (42.4%), M = 57 (57.6%); average age 44.9 ± 8.65; average height 174.8 ± 9.8 cm, average weight 82.4 ± 10.6 kg; average BMI 27.0 ± 1.95 kg/m2). In the newly involved recreational group (REC) there were 77 participants (F = 67 (87%), M = 10 (13%), average age 50.6 ± 6.0; average height 168.0 ± 7.4 cm, average weight 75.1 ± 9.4 kg; average BMI 26.6 ± 2.1 kg/m2). In the professional competing athletes group (PRO), there were 38 participants (F = 5 (13.2%), M = 33 (86.8%), average age 31.2 ± 8.6; average height 185.8 ± 7.0 cm, average weight 85.5 ± 12.2 kg; average BMI 24.7 ± 2.7 kg/m2). In the regularly moderate active group (ACT) there were 62 participants (F = 24 (38.7%), M = 38 (61.3%), average age 40.9 ± 8.4; average height 176.7 ± 10.1 cm, average weight 76.3 ± 16.2 kg; average BMI 24.2 ± 3.3 kg/m2). All participants reported their prior and current activity level, training intensity where it applies, possible prior injuries and competing status for competing group, as well as their competing sport. Minimal requirements for each group were as follows: professional athletes were either single or team categorized competing athletes in their respective sports (athletics, martial arts, football, basketball, handball, water polo, rowing, hockey), with structured and organized trainings on average one training per day. In the regularly moderate active group participants have had mostly supervised trainings 2–3 times per week regularly, and for minimum of three years prior to entering this study, but most of them were life-long exercisers. In the newly involved recreational group participants have started with regular, supervised moderate physical activity 2 times per week, and they were included in this study after the first exercise season (9 months of moderate, regular physical activity with 1 month of winter break and two months of summer break), after being sedentary and inactive for at least 5 years prior to starting with moderate physical activity. Lastly, the inactive group consisted of sedentary population that has no regular physical activity in their everyday lives, for at least last five years, most of them even longer.

### Experimental protocol

All participants underwent the same initial protocol during their yearly check-ups with their medical doctor, voluntarily entering the study, filling in the questionnaire, signing the informed consent and having their morphological characteristics determined. Morphological characteristics included body mass, height, and body mass index (BMI). Following the initial questionnaire, an informed consent was signed, and morphological characteristics were determined, following with collection of dry blood spots (DBS) from each participant. Following the DBC collection protocol, selected finger was wiped with ethanol and air dried. MD performed skin puncturing with a 21 G BD contact-activated lancet (BD, Franklin Lakes, NJ, USA). After the blood drop was formed, it was transferred to form a blood spot on Whatman Human ID Bloodstain Cards, Cat. No. WB100014 (GE Healthcare, Anaheim, CA, USA). The blood spots on collection cards were left to air dry at room temperature for next two hours, afterwards being stored in air-tight zipped bags, containing desiccant at -20 °C [23].

IgG N-glycan analysis was conducted in accordance with the protocol outlined by Šimunović et al. [23], incorporating slight adjustments. Initially, DBS samples were cut into smaller fragments, placed in a 96-well collection plate, and gently shaken for three hours at room temperature with 800 µL 1x phosphate buffer saline (1× PBS, prepared in-house). The isolation of IgG from DBS was performed using CIM® r-Protein G LLD 0.2 mL Monolithic 96-well Plate (2 μm channels) (BIA separations, Ajdovščina, Slovenia) following an optimized high-throughput method [24]. In summary, IgG was eluted with 0.1 M formic acid (prepared in-house) and promptly neutralized with 1 M ammonium bicarbonate (prepared in-house). The resulting IgG eluate (600 µL) underwent overnight drying in a vacuum concentrator. The dried IgG was subsequently desalted with 800 µL methanol (Honeywell, Charlotte, NC, USA) cooled to − 20 ◦C. After resuspension and centrifugation at 2000× g for the duration of 15 min, 78 µL of the supernatant was carefully extracted. This process was repeated with an additional 800 µL of cold methanol, and the protein precipitate was left to dry in a vacuum concentrator for two hours. The enzymatic release of N-glycans from the dried and desalted IgG employed the specific enzyme PNGase F, and the resulting free IgG N-glycans were subsequently labelled with 2-aminobenzamide (2-AB). The fluorescently labelled IgG N-glycans were then subjected to analysis using hydrophilic interaction liquid chromatography (HILIC) ultraperformance liquid chromatography (UPLC) [25]. The obtained chromatograms were manually integrated and categorized into 24 glycan peaks (GP). A comprehensive list of IgG N-glycan structures corresponding to individual glycan peaks is available in Supplementary Fig. 1.

### Statistical analysis

To make the glycan measurements comparable across the samples, normalization was performed by expressing the amount of glycans in each peak as % of total integrated area. Next, glycan measurements were log-transformed and batch correction was applied using the “ComBat()” function within the “sva” package in R [26], where the plate was modelled as a batch covariate. Once the estimated batch effects were subtracted from log-transformed values, the values were exponentiated to obtain corrected measurements on the original scale and GlycanAge was derived [14, 15]. Differences in glycan measurements (GP1-GP24) and GlycanAge between groups were determined in pairwise manner (Inactive vs. Active; Inactive vs. Recreational; Inactive vs. Professional; Recreational vs. Active; Active vs. Professional) using regression model where glycan values were included as dependent variable and group was modelled as independent variable with age and sex included as additional covariates. An inverse transformation of ranks to normality was applied to glycan values prior to statistical testing. Differences in GlycanAge were also determined in sex-stratified manner. To control for multiple testing, we performed a correction using the “p.adjust()” function in R, which implements the Benjamini–Hochberg procedure for controlling the false discovery rate (method = “fdr”). An adjusted p-value of < 0.05 was considered as significant. Data analysis and visualization were carried out using R software (version 4.3.0) [27].

## Results

The glycosylation of immunoglobulin G was analyzed in 276 healthy participants with different intensity level of physical activity to investigate the impact of exercise intensity on the composition of IgG N-glycans. The N-glycome composition was used to derive the GlycanAge index for each participant.

**Fig. 1:**
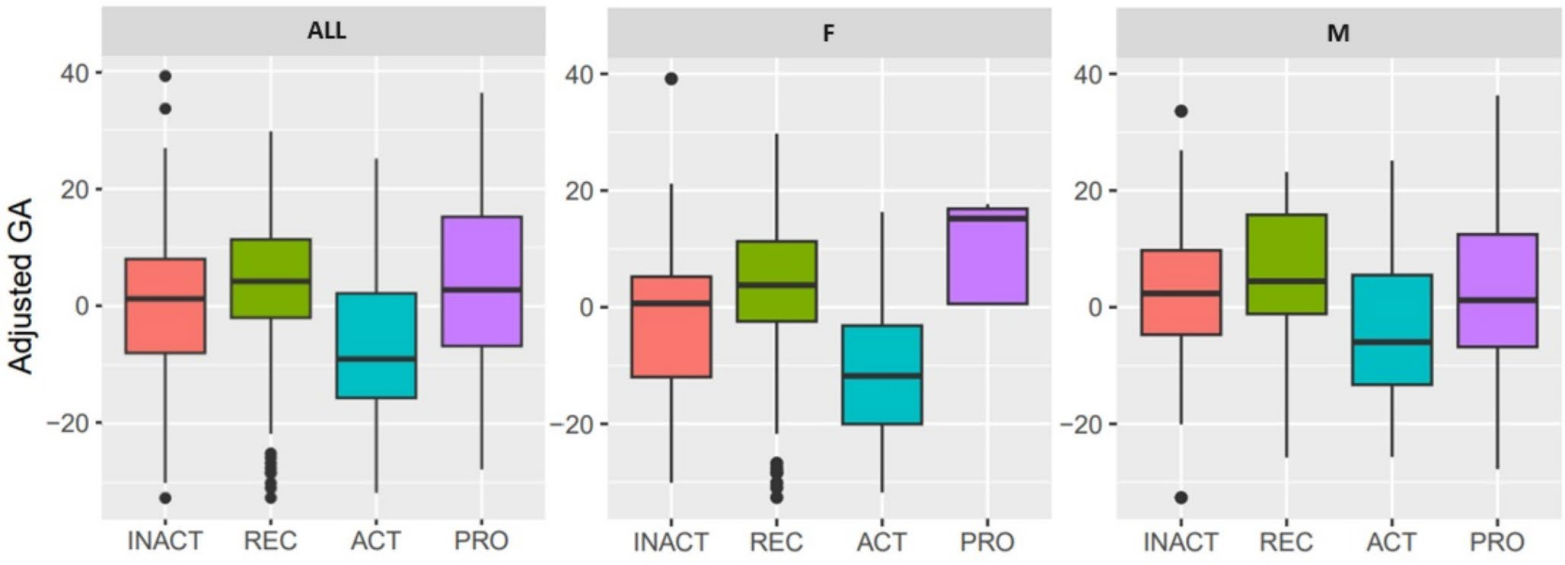
Box plots showing differences in age adjusted GlycanAge between different intensity groups for all and between genders (F/M). Residual GlycanAge values are plotted after adjusting GlycanAge values for chronological age (red box: inactive group, green box: recreational group, blue box: active group, purple box: professional group)

Participants in the active group, exercising regularly for at least three years, most of them even longer, had significantly lower GlycanAge when compared to the participants in the inactive group (β = -7.437, p.adj = 7.85E-03). Furthermore, differences were even more pronounced when stratified for sex, females in the active group had even lower GlycanAge when compared to the females in the inactive group (β = -9.762, p.adj = 4.68E-02), while the male counterparts had nominally significant effect with same trends, when compared to the male participants in the inactive group (β = -5.911, *p* = 3.86E-02). Participants in the professional group had a nominally significant effect in the opposite direction, having higher GlycanAge, when compared to the participants in the active group, (β = 7.546, *p* = 3.20E-02). Following the same trend as mentioned in the pairwise comparison between the active and inactive group; females in the professional group had higher GlycanAge when compared to the females in the active group (β = 20.206, p.adj = 2.71E-02), while male counterparts in the professional group lacked the significant results, when compared to the male participants in the active group (β = 4.388, *p* = 4.63E-01). Lastly, participants in the active group had nominally lower GlycanAge when compared to the participants in the inactive group (β = -8.278, *p* = 1.25E-02, p.adj = 5.46E-02). Following the trends mentioned above, females in the active group had nominally lower GlycanAge when compared to the females in the recreational group (β = -8.239, *p* = 4.92E-02), while male counterparts in the active group lacked the significant results, when compared to the male participants in the recreational group (β = -6.138, *p* = 2.72E-01) (Table 1; Fig. 1).

**Table 1:** Pairwise comparisons and effect estimate of exercise on GlycanAge between different intensity groups, after adjusting for chronological age and sex (ALL), and pairwise comparison and effect estimate of exercise on GlycanAge between different intensity groups in two gender groups (F/M) after adjusting for chronological age. Effect size, standard error (SE), *p* value (p). P-values are adjusted for multiple testing using Benjamini-Hochberg procedure to control for false discovery rate (FDR)

Pairwise comparison	ALL/sex-specific	Effect	SE	p	p.adjusted
Inactive vs. active	ALL	-7.44	2.24	1.10E-03	**7.85E-03**
Inactive vs. active	F	-9.76	3.70	1.04E-02	**4.68E-02**
Inactive vs. active	M	-5.91	2.82	**3.86E-02**	1.24E-01
Active vs. professional	ALL	7.55	3.47	**3.20E-02**	1.08E-01
Active vs. professional	F	20.21	6.66	5.41E-03	**2.71E-02**
Active vs. professional	M	4.39	4.07	2.85E-01	4.63E-01
Recreational vs. active	ALL	-8.28	3.27	**1.25E-02**	5.46E-02
Recreational vs. active	F	-8.24	4.13	**4.92E-02**	1.54E-01
Recreational vs. active	M	-6.14	5.52	2.72E-01	4.48E-01
Inactive vs. recreational	ALL	2.82	2.52	2.66E-01	4.46E-01
Inactive vs. recreational	F	2.92	3.10	3.48E-01	5.28E-01
Inactive vs. recreational	M	0.88	4.65	8.50E-01	9.18E-01
Inactive vs. professional	ALL	-0.69	3.31	8.35E-01	9.09E-01
Inactive vs. professional	F	7.59	7.40	3.11E-01	4.94E-01
Inactive vs. professional	M	-2.04	3.78	5.91E-01	7.18E-01

Changes in the abundance of specific glycan peaks are observed between different intensity exercise groups and inactive group. When comparing active and inactive groups, GP3, GP5, GP11, GP17, GP19, GP20, GP21 and GP24 were significantly decreased, while GP14 was significantly increased; GP4 was nominally decreased, while GP8 was nominally increased. When comparing active and professional groups, GP4 was significantly increased, while GP15 was significantly decreased; additionally, GP10, GP11, GP14 and GP16 were nominally decreased. In the comparison of active and recreational group, GP3, GP5 and GP11 were significantly decreased, while GP21 was nominally increased. Comparing the recreational group to the inactive group, GP5, GP17, GP20 and GP21 were significantly decreased. Lastly, comparing professionals to the inactive group we have observed following differences: GP5, GP11, GP13, GP17, GP19, GP20, GP21 and GP24 were significantly decreased, GP8 was significantly increased; while GP3 and GP16 were nominally decreased (effect estimates and p.adj values are presented in Table 2, list of IgG N-glycan structures corresponding to individual glycan peaks is available in Supplementary Fig. 1).

**Table 2:** Pairwise comparisons and effect estimate of exercise on GP1-GP24 between groups, after adjusting for age and sex. Effect size, standard error (SE), p value (p). P-values are adjusted for multiple testing using Benjamini–Hochberg procedure to control for false discovery rate (FDR). Only data with nominally significant and significant p values is presented. Full data set is available in Supplementary Table 1

Pairwise comparison	trait	Effect	SE	p	p.adjusted
Inactive vs. active	GP5	-1.25	0.14	3.39E-16	**4.58E-14**
Inactive vs. active	GP20	-0.75	0.15	2.23E-06	**5.01E-05**
Inactive vs. active	GP3	-0.69	0.15	5.00E-06	**7.50E-05**
Inactive vs. active	GP24	-0.73	0.15	4.72E-06	**7.50E-05**
Inactive vs. active	GP11	-0.66	0.14	5.77E-06	**7.79E-05**
Inactive vs. active	GP21	-0.65	0.14	1.16E-05	**1.42E-04**
Inactive vs. active	GP17	-0.66	0.15	2.07E-05	**2.33E-04**
Inactive vs. active	GP19	-0.56	0.15	3.62E-04	**3.05E-03**
Inactive vs. active	GP14	0.39	0.12	2.24E-03	**1.26E-02**
Inactive vs. active	GP4	-0.33	0.14	**1.70E-02**	6.95E-02
Inactive vs. active	GP8	0.35	0.16	**2.69E-02**	1.01E-01
Active vs. professional	GP4	0.55	0.17	2.02E-03	**1.23E-02**
Active vs. professional	GP15	-0.59	0.19	2.10E-03	**1.23E-02**
Active vs. professional	GP11	-0.45	0.19	**1.68E-02**	6.95E-02
Active vs. professional	GP16	-0.51	0.22	**2.45E-02**	9.47E-02
Active vs. professional	GP10	-0.48	0.22	**3.13E-02**	1.08E-01
Active vs. professional	GP14	-0.35	0.16	**3.00E-02**	1.08E-01
Recreational vs. active	GP5	-0.65	0.17	2.57E-04	**2.32E-03**
Recreational vs. active	GP3	-0.55	0.20	7.83E-03	**3.77E-02**
Recreational vs. active	GP11	-0.51	0.19	9.56E-03	**4.45E-02**
Recreational vs. active	GP21	0.32	0.15	**3.73E-02**	1.23E-01
Inactive vs. recreational	GP21	-0.77	0.15	6.69E-07	**1.81E-05**
Inactive vs. recreational	GP17	-0.76	0.16	3.00E-06	**5.79E-05**
Inactive vs. recreational	GP5	-0.49	0.16	1.87E-03	**1.20E-02**
Inactive vs. recreational	GP20	-0.48	0.17	5.30E-03	**2.71E-02**
Inactive vs. professional	GP5	-1.32	0.21	4.78E-09	**3.23E-07**
Inactive vs. professional	GP11	-1.21	0.20	1.88E-08	**8.44E-07**
Inactive vs. professional	GP20	-1.32	0.25	3.58E-07	**1.21E-05**
Inactive vs. professional	GP21	-0.96	0.24	7.31E-05	**7.59E-04**
Inactive vs. professional	GP17	-0.98	0.24	9.54E-05	**9.19E-04**
Inactive vs. professional	GP24	-0.85	0.23	4.31E-04	**3.42E-03**
Inactive vs. professional	GP8	0.79	0.22	4.91E-04	**3.68E-03**
Inactive vs. professional	GP13	-0.77	0.24	1.63E-03	**1.10E-02**
Inactive vs. professional	GP19	-0.75	0.25	2.71E-03	**1.46E-02**
Inactive vs. professional	GP3	-0.50	0.21	**2.14E-02**	8.48E-02
Inactive vs. professional	GP16	-0.51	0.24	**3.16E-02**	1.08E-01

## Discussion

In this study, we investigated the effects of exercise intensity on the GlycanAge index, biomarker of aging, in several groups exercising in different manners. The IgG N-glycans reflect pro- and anti-inflammatory events happening throughout one’s organism, making them very responsive markers of different types of interventions. The main result of our study shows that the active group has 7.4 GlycanAge years less when compared to the inactive group, tremendously underlying the importance of regular physical activity throughout one’s lifetime. When stratifying by sex, the results are even more prominent. Females in the active group exhibit a younger GlycanAge profile, almost for a full decade younger compared to their counterparts in the inactive group, while males are nominally younger by almost 6 GlycanAge years compared to their respective counterparts. It is known that regular physical activity has the beneficial effect on chronic inflammation, as cross-sectional study has reported an inverse association between exercise and inflammatory blood biomarkers [28].

According to data from the European Health Interview Study (EHIS) in Croatia in 2019, almost three quarters of the general population is overweight (BMI > 25) or obese (BMI > 30), accounting for 49.5% and 23.7%, respectively. Only a little over one quarter falls into the normal and underweight categories, comprising 26.6% and 0.3%, respectively, painting a gloomy picture. Furthermore, 2010 data shows that 66% male and 75% female population with a stroke related diagnosis are overweight. Similarly, 78% of males and 74% of females with a hypertension related diagnosis are overweight, as well as 79% of male and 84% of female population with a T2D diagnosis are overweight. Additionally, only 19.5% of the population is physically active for at least 150 min weekly, as recommended by the WHO, while 36% of the population spends 7 or more hours daily in a sedentary manner, excluding sleeping [29]. Metabolic syndrome, a major public health concern, is defined by intertwined risk factors, including central obesity, hypertension, abnormalities in blood sugar stability, as well as an imbalance of lipids. Visceral adipose tissue, with its significant metabolic activity and secretion of pro-inflammatory cytokines and adipokines, plays a crucial role in the pro-inflammatory insulin resistance state. Therefore, changing body composition is of utmost importance. Regular moderate exercise training over time induces changes in body composition, and reduced body fat, ultimately diminishing the pro-inflammatory state, shifting it towards an anti-inflammatory status [30].

The reduction of glycans with the bisecting N-acetyl glucosamine (GlcNAc) is associated with decreased inflammatory potential of IgG, while their increased abundance is associated with T2D and with a higher cardiovascular risk. In the active group, four out of nine glycan peaks containing bisected GlcNAc structures are significantly lower than in the inactive group. An increase in digalactosylated and sialylated glycans is associated with anti-inflammatory IgG potential, while an increase in agalactosylated and asialylated structures is associated with a pro-inflammatory status.

Professional competing athletes train at higher intensities, with higher training frequency, resulting in less time in between to recover. Compared to the active group that practices moderate exercise, our results show that the group of professional athletes has 7.6 more GlycanAge years. Similarly, as mentioned above, when stratified by sex, females in the professional group show a dramatically older GlycanAge profile, two full decades older, compared to their counterparts in the active group. It is important to underline that there is a very limited number of professional female athletes in this study (N = 5), but somewhat similar results were reported in the study performed by Sarin et al. [20]. In the aforementioned study, Sarin et al. investigated the impact of prolonged intensive exercise training, coupled with a low-calorie diet in 42 healthy women (age 27.5 ± 4.0 years, body mass index 23.4 ± 1.7 kg/m2). After 4 months of intervention, resulting in intense weight loss, the test group had a significant pro-inflammatory IgG N-glycan profile. This was characterized by a decrease in glycan peaks containing digalactosylated and sialylated glycans, while glycans with bisecting GlcNAc, agalactosylated, and monogalactosylated glycans were significantly increased when compared to the control group.

Jurić et al. reported a significant effect of estradiol on biological age in females, which could be one of the reasons why professional female athletes have a significantly higher GlycanAge than their counterparts, as professional athletes often have a very low percentage of body fat, which is known to potentially interfere with the menstrual cycle [15]. They showed that the deprivation of gonadal hormones results in a median increase in GlycanAge for 9.1 years, suggesting a significant influence of hormone levels on IgG glycans. On the other hand, in the male population, this trend of a significantly increased GlycanAge between professional athletes and the active male population was not observed. Tijardović et al. did not report any significant pro-inflammatory increase in IgG N-glycans in their repeated sprint training male cohort (age 20.0 ± 1.0 years, height 181 ± 4.0 cm, weight 77.7 ± 6.0 kg) [19]. In our study, male professional athletes did not have such a prominent effect, showing no significant differences compared to their respective counterparts in the active group.

These results, combined with ours, imply that intense physical activity has the adverse effects on both male and female population. Unlike regular moderate physical activity, exercising at high intensities causes oxidative damage, triggering an immediate immunological response. High intensities stimulate oxygen consumption, leading to the formation of reactive oxygen species (ROS) produced by mitochondrial activity and increased aerobic respiration. Oxidative stress occurs when there is an imbalance between ROS formation and antioxidant response [30]. Furthermore, chronic exhaustive training is considered the cause of an imbalance between Th1 and Th2 profiles, with Th2 becoming predominant, resulting in cellular immunosuppression, inflammation, and increased susceptibility to infections [31, 32]. Overtraining syndrome (OTS) in professional athletes leads to an overall degradation of performance and results, with the immune system being particularly compromised. To prevent OTS, monitoring of exercise training load and other stressors is necessary to detect the development of OTS in a timely manner [33].

Regular moderate exercise training has beneficial effects over time. When comparing a group of recreational exercisers to the inactive group, we did not observe any significant results, mainly to the underlying effect of obesity in the beforementioned group. The lack of physical activity, coupled with an unhealthy diet and a sedentary lifestyle, is likely to increase bodyweight. Almost three quarters of our general population are overweight and obese [29], so many individuals introduce moderate exercise at the latter stages of body weight gain. Higher levels of body fat are associated with a pro-inflammatory IgG N-glycan profile, while weight loss and the reduction of body fat substantially affect IgG N-glycosylation, resulting in an anti-inflammatory shift and ultimately leading to reduced biological age and lower GlycanAge index [16–18]. As our study populations were smaller in number, without optimal gender ration, further research would be beneficial to verify our results. Additionally, longitudinal studies involving inactive, active, and professional groups would further expand the knowledge of the impact of training frequency and intensity on the inflammatory status of athletes. High amount of sedentary time, coupled with low amount of moderate to high intensity physical activity and low CFR are associated with poor metabolic health and higher metabolic syndrome and T2D risk [34]. Even the increase from low to moderate amount of moderate physical activity, while reducing sedentary time will increase CRF, resulting in reduced risks of cardiometabolic disorder occurrence, leading to a healthier life.

## Conclusion

In summary, our results are reporting a decreased GlycanAge index in an active group of moderately exercising population, while both the inactive group and professional athletes group, when compared to the active group, demonstrate an increased GlycanAge index. Our findings support the notion that moderate physical exercise is a widely available, anti-inflammatory strategy with mild to no side effects for the general population. Physical activity has great potential to mitigate growing issues related to obesity and sedentary lifestyle, which are directly linked to an increased inflammatory status and the development of non-communicable diseases.

### Electronic supplementary material

Below is the link to the electronic supplementary material.

Supplementary Material 1

## Data Availability

The data sets generated during and/or analyzed during the current study are available from the corresponding author on reasonable request.
