# Correction to: Regular moderate physical exercise decreases Glycan Age index of biological age and reduces inflammatory potential of Immunoglobulin G

**DOI:** 10.1007/s10719-024-10146-x

**Published:** 2024-01-11

**Authors:** Nina Šimunić-Briški, Vedran Dukarić, Mateja Očić, Tomislav Madžar, Martina Vinicki, Azra Frkatović-Hodžić, Damir Knjaz, Gordan Lauc

**Affiliations:** 1 Genos Ltd., Zagreb, 10000 Croatia; 2 Faculty of Kinesiology, University of Zagreb, Zagreb, 10000 Croatia; 3 Faculty of Pharmacy and Biochemistry, University of Zagreb, Zagreb, 10000 Croatia; 4 Vaš Pregled Sports and Occupation Medicine Polyclinic, Zagreb, 10000 Croatia; 5 University of Applied Health Sciences, Zagreb, 10000 Croatia

**Correction to: Glycoconjugate Journal (2023)**

10.1007/s10719-023-10144-5

The article “Regular moderate physical exercise decreases Glycan Age index of biological age and reduces inflammatory potential of Immunoglobulin G”, Nina Šimunić-Briški, Vedran Dukarić, Mateja Očić, Tomislav Madžar, Martina Vinicki, Azra Frkatović-Hodžić, Damir Knjaz and Gordan Lauc was originally published Online First without Open Access. The author decided to opt for Open Choice and to make the article an Open Access publication. Therefore, the copyright of the article has been changed to © The Author(s) 2023 and the article is forthwith distributed under the terms of the Creative Commons Attribution 4.0 International License, which permits use, sharing, adaptation, distribution and reproduction in any medium or format, as long as you give appropriate credit to the original author(s) and the source, provide a link to the Creative Commons licence, and indicate if changes were made. The images or other third party material in this article are included in the article’s Creative Commons licence, unless indicated otherwise in a credit line to the material. If material is not included in the article’s Creative Commons licence and your intended use is not permitted by statutory regulation or exceeds the permitted use, you will need to obtain permission directly from the copyright holder. To view a copy of this licence, visit http://creativecommons.org/licenses/by/4.0/.

The original article has been corrected.

