# Supplementary material for: Regular moderate physical exercise decreases Glycan Age index of biological age and reduces inflammatory potential of Immunoglobulin G
Source: Glycoconj J. 2023 Dec 26;41(1):67–76. doi: 10.1007/s10719-023-10144-5 (PMC10957704; doi:10.1007/s10719-023-10144-5)
Supplement: Supplementary file 1 — Supplementary Material 1 [file 10719_2023_10144_MOESM1_ESM.docx]

Supplementary files

| Pairwise comparison | trait | Effect | SE | p | p.adjusted |
| --- | --- | --- | --- | --- | --- |
| inactive vs active | GP1 | -0.16665 | 0.14184 | 2.42E-01 | 4.24E-01 |
| inactive vs active | GP2 | 0.03529 | 0.13342 | 7.92E-01 | 8.82E-01 |
| inactive vs active | GP3 | -0.69326 | 0.14661 | 5.00E-06 | **7.50E-05** |
| inactive vs active | GP4 | -0.32798 | 0.13594 | **1.70E-02** | 6.95E-02 |
| inactive vs active | GP5 | -1.24883 | 0.13691 | 3.39E-16 | **4.58E-14** |
| inactive vs active | GP6 | -0.12008 | 0.1319 | 3.64E-01 | 5.42E-01 |
| inactive vs active | GP7 | 0.14739 | 0.14883 | 3.24E-01 | 5.02E-01 |
| inactive vs active | GP8 | 0.35167 | 0.15738 | **2.69E-02** | 1.01E-01 |
| inactive vs active | GP9 | 0.02672 | 0.16388 | 8.71E-01 | 9.29E-01 |
| inactive vs active | GP10 | 0.14114 | 0.15544 | 3.65E-01 | 5.42E-01 |
| inactive vs active | GP11 | -0.65804 | 0.14015 | 5.77E-06 | **7.79E-05** |
| inactive vs active | GP12 | 0.21921 | 0.15481 | 1.59E-01 | 3.30E-01 |
| inactive vs active | GP13 | -0.19129 | 0.15125 | 2.08E-01 | 4.01E-01 |
| inactive vs active | GP14 | 0.38654 | 0.12438 | 2.24E-03 | **1.26E-02** |
| inactive vs active | GP15 | 0.18794 | 0.14865 | 2.08E-01 | 4.01E-01 |
| inactive vs active | GP16 | 0.04012 | 0.15579 | 7.97E-01 | 8.82E-01 |
| inactive vs active | GP17 | -0.66045 | 0.15043 | 2.07E-05 | **2.33E-04** |
| inactive vs active | GP18 | 0.22943 | 0.12892 | 7.71E-02 | 1.96E-01 |
| inactive vs active | GP19 | -0.557 | 0.15277 | 3.62E-04 | **3.05E-03** |
| inactive vs active | GP20 | -0.75195 | 0.15303 | 2.23E-06 | **5.01E-05** |
| inactive vs active | GP21 | -0.64897 | 0.14322 | 1.16E-05 | **1.42E-04** |
| inactive vs active | GP22 | -0.22301 | 0.15992 | 1.65E-01 | 3.38E-01 |
| inactive vs active | GP23 | -0.13163 | 0.15497 | 3.97E-01 | 5.52E-01 |
| inactive vs active | GP24 | -0.7251 | 0.15291 | 4.72E-06 | **7.50E-05** |
| active vs professional | GP1 | 0.22731 | 0.18975 | 2.34E-01 | 4.21E-01 |
| active vs professional | GP2 | 0.12726 | 0.23352 | 5.87E-01 | 7.18E-01 |
| active vs professional | GP3 | 0.28245 | 0.17589 | 1.12E-01 | 2.63E-01 |
| active vs professional | GP4 | 0.55121 | 0.17364 | 2.02E-03 | **1.23E-02** |
| active vs professional | GP5 | 0.06153 | 0.17492 | 7.26E-01 | 8.17E-01 |
| active vs professional | GP6 | 0.17964 | 0.21039 | 3.95E-01 | 5.52E-01 |
| active vs professional | GP7 | 0.00314 | 0.2517 | 9.90E-01 | 9.90E-01 |
| active vs professional | GP8 | 0.22809 | 0.20448 | 2.67E-01 | 4.46E-01 |
| active vs professional | GP9 | -0.26938 | 0.22056 | 2.25E-01 | 4.17E-01 |
| active vs professional | GP10 | -0.47871 | 0.21906 | **3.13E-02** | 1.08E-01 |
| active vs professional | GP11 | -0.4503 | 0.1851 | **1.68E-02** | 6.95E-02 |
| active vs professional | GP12 | -0.24191 | 0.24117 | 3.18E-01 | 5.00E-01 |
| active vs professional | GP13 | -0.42824 | 0.21601 | **5.03E-02** | 1.54E-01 |
| active vs professional | GP14 | -0.3535 | 0.1605 | **3.00E-02** | 1.08E-01 |
| active vs professional | GP15 | -0.5923 | 0.18735 | 2.10E-03 | **1.23E-02** |
| active vs professional | GP16 | -0.50157 | 0.21955 | **2.45E-02** | 9.47E-02 |
| active vs professional | GP17 | -0.1124 | 0.22735 | 6.22E-01 | 7.30E-01 |
| active vs professional | GP18 | -0.33653 | 0.17329 | 5.51E-02 | 1.62E-01 |
| active vs professional | GP19 | 0.14407 | 0.22531 | 5.24E-01 | 6.65E-01 |
| active vs professional | GP20 | -0.27869 | 0.20472 | 1.77E-01 | 3.56E-01 |
| active vs professional | GP21 | -0.15948 | 0.20244 | 4.33E-01 | 5.92E-01 |
| active vs professional | GP22 | 0.11073 | 0.2219 | 6.19E-01 | 7.30E-01 |
| active vs professional | GP23 | 0.27922 | 0.21412 | 1.95E-01 | 3.88E-01 |
| active vs professional | GP24 | 0.15171 | 0.20275 | 4.56E-01 | 6.04E-01 |
| recreational vs active | GP1 | -0.33819 | 0.23474 | 1.52E-01 | 3.21E-01 |
| recreational vs active | GP2 | 0.17145 | 0.22065 | 4.38E-01 | 5.92E-01 |
| recreational vs active | GP3 | -0.54708 | 0.20263 | 7.83E-03 | **3.77E-02** |
| recreational vs active | GP4 | -0.33008 | 0.20698 | 1.13E-01 | 2.63E-01 |
| recreational vs active | GP5 | -0.65111 | 0.17343 | 2.57E-04 | **2.32E-03** |
| recreational vs active | GP6 | -0.37805 | 0.19748 | 5.77E-02 | 1.66E-01 |
| recreational vs active | GP7 | 0.39675 | 0.23099 | 8.82E-02 | 2.13E-01 |
| recreational vs active | GP8 | 0.18549 | 0.20732 | 3.73E-01 | 5.47E-01 |
| recreational vs active | GP9 | 0.10424 | 0.23908 | 6.64E-01 | 7.61E-01 |
| recreational vs active | GP10 | -0.33585 | 0.21939 | 1.28E-01 | 2.88E-01 |
| recreational vs active | GP11 | -0.51049 | 0.19417 | 9.56E-03 | **4.45E-02** |
| recreational vs active | GP12 | 0.34815 | 0.2211 | 1.18E-01 | 2.69E-01 |
| recreational vs active | GP13 | 0.14823 | 0.21493 | 4.92E-01 | 6.44E-01 |
| recreational vs active | GP14 | 0.35485 | 0.19491 | 7.09E-02 | 1.84E-01 |
| recreational vs active | GP15 | -0.04731 | 0.2091 | 8.21E-01 | 9.01E-01 |
| recreational vs active | GP16 | -0.01868 | 0.23184 | 9.36E-01 | 9.64E-01 |
| recreational vs active | GP17 | 0.30955 | 0.17848 | 8.51E-02 | 2.09E-01 |
| recreational vs active | GP18 | 0.39166 | 0.20821 | 6.21E-02 | 1.75E-01 |
| recreational vs active | GP19 | -0.24131 | 0.21626 | 2.66E-01 | 4.46E-01 |
| recreational vs active | GP20 | 0.00851 | 0.1774 | 9.62E-01 | 9.76E-01 |
| recreational vs active | GP21 | 0.31537 | 0.14995 | **3.73E-02** | 1.23E-01 |
| recreational vs active | GP22 | 0.22004 | 0.23255 | 3.46E-01 | 5.28E-01 |
| recreational vs active | GP23 | 0.24954 | 0.20897 | 2.35E-01 | 4.21E-01 |
| recreational vs active | GP24 | -0.40838 | 0.22096 | 6.68E-02 | 1.80E-01 |
| inactive vs recreational | GP1 | 0.22168 | 0.1822 | 2.25E-01 | 4.17E-01 |
| inactive vs recreational | GP2 | -0.07958 | 0.15968 | 6.19E-01 | 7.30E-01 |
| inactive vs recreational | GP3 | -0.06847 | 0.17267 | 6.92E-01 | 7.85E-01 |
| inactive vs recreational | GP4 | 0.18209 | 0.17221 | 2.92E-01 | 4.69E-01 |
| inactive vs recreational | GP5 | -0.49394 | 0.15634 | 1.87E-03 | **1.20E-02** |
| inactive vs recreational | GP6 | 0.29501 | 0.15118 | 5.26E-02 | 1.58E-01 |
| inactive vs recreational | GP7 | -0.1503 | 0.1742 | 3.89E-01 | 5.52E-01 |
| inactive vs recreational | GP8 | -0.07387 | 0.17036 | 6.65E-01 | 7.61E-01 |
| inactive vs recreational | GP9 | 0.0179 | 0.18825 | 9.24E-01 | 9.60E-01 |
| inactive vs recreational | GP10 | 0.248 | 0.16988 | 1.46E-01 | 3.13E-01 |
| inactive vs recreational | GP11 | 0.01513 | 0.16007 | 9.25E-01 | 9.60E-01 |
| inactive vs recreational | GP12 | -0.15549 | 0.17666 | 3.80E-01 | 5.52E-01 |
| inactive vs recreational | GP13 | -0.22985 | 0.18322 | 2.11E-01 | 4.02E-01 |
| inactive vs recreational | GP14 | -0.14148 | 0.16239 | 3.85E-01 | 5.52E-01 |
| inactive vs recreational | GP15 | 0.11445 | 0.17788 | 5.21E-01 | 6.65E-01 |
| inactive vs recreational | GP16 | 0.11777 | 0.18638 | 5.28E-01 | 6.65E-01 |
| inactive vs recreational | GP17 | -0.76404 | 0.15818 | 3.00E-06 | **5.79E-05** |
| inactive vs recreational | GP18 | -0.24497 | 0.16629 | 1.43E-01 | 3.10E-01 |
| inactive vs recreational | GP19 | -0.02897 | 0.18194 | 8.74E-01 | 9.29E-01 |
| inactive vs recreational | GP20 | -0.48016 | 0.17 | 5.30E-03 | **2.71E-02** |
| inactive vs recreational | GP21 | -0.77094 | 0.14935 | 6.69E-07 | **1.81E-05** |
| inactive vs recreational | GP22 | -0.20614 | 0.18173 | 2.58E-01 | 4.46E-01 |
| inactive vs recreational | GP23 | -0.30457 | 0.16609 | 6.84E-02 | 1.81E-01 |
| inactive vs recreational | GP24 | -0.09255 | 0.18604 | 6.19E-01 | 7.30E-01 |
| inactive vs professional | GP1 | 0.01989 | 0.20236 | 9.22E-01 | 9.60E-01 |
| inactive vs professional | GP2 | 0.32001 | 0.21012 | 1.30E-01 | 2.88E-01 |
| inactive vs professional | GP3 | -0.49713 | 0.21346 | **2.14E-02** | 8.48E-02 |
| inactive vs professional | GP4 | 0.12479 | 0.19921 | 5.32E-01 | 6.65E-01 |
| inactive vs professional | GP5 | -1.31974 | 0.21062 | 4.78E-09 | **3.23E-07** |
| inactive vs professional | GP6 | 0.12452 | 0.20228 | 5.39E-01 | 6.68E-01 |
| inactive vs professional | GP7 | 0.17969 | 0.23104 | 4.38E-01 | 5.92E-01 |
| inactive vs professional | GP8 | 0.78669 | 0.22014 | 4.91E-04 | **3.68E-03** |
| inactive vs professional | GP9 | -0.2737 | 0.23044 | 2.37E-01 | 4.21E-01 |
| inactive vs professional | GP10 | -0.13965 | 0.22218 | 5.31E-01 | 6.65E-01 |
| inactive vs professional | GP11 | -1.21428 | 0.20283 | 1.88E-08 | **8.44E-07** |
| inactive vs professional | GP12 | -0.01427 | 0.23563 | 9.52E-01 | 9.73E-01 |
| inactive vs professional | GP13 | -0.76678 | 0.2384 | 1.63E-03 | **1.10E-02** |
| inactive vs professional | GP14 | 0.08341 | 0.18697 | 6.56E-01 | 7.61E-01 |
| inactive vs professional | GP15 | -0.38513 | 0.22167 | 8.46E-02 | 2.09E-01 |
| inactive vs professional | GP16 | -0.51002 | 0.23475 | **3.16E-02** | 1.08E-01 |
| inactive vs professional | GP17 | -0.97567 | 0.24244 | 9.54E-05 | **9.19E-04** |
| inactive vs professional | GP18 | -0.14359 | 0.19071 | 4.53E-01 | 6.04E-01 |
| inactive vs professional | GP19 | -0.75077 | 0.24562 | 2.71E-03 | **1.46E-02** |
| inactive vs professional | GP20 | -1.32353 | 0.24695 | 3.58E-07 | **1.21E-05** |
| inactive vs professional | GP21 | -0.96477 | 0.23564 | 7.31E-05 | **7.59E-04** |
| inactive vs professional | GP22 | -0.4189 | 0.22552 | 6.54E-02 | 1.80E-01 |
| inactive vs professional | GP23 | -0.00486 | 0.22086 | 9.82E-01 | 9.90E-01 |
| inactive vs professional | GP24 | -0.84593 | 0.23428 | 4.31E-04 | **3.42E-03** |

Supplementary table 1. Pairwise comparisons and effect estimates of exercise on GP1-GP24 between groups, after adjusting for age and sex. Effect size, standard error (SE), p value (p). P-values are adjusted for multiple testing using Benjamini–Hochberg procedure to control for false-discovery rate (FDR). Only nominally significant and significant p values are presented.


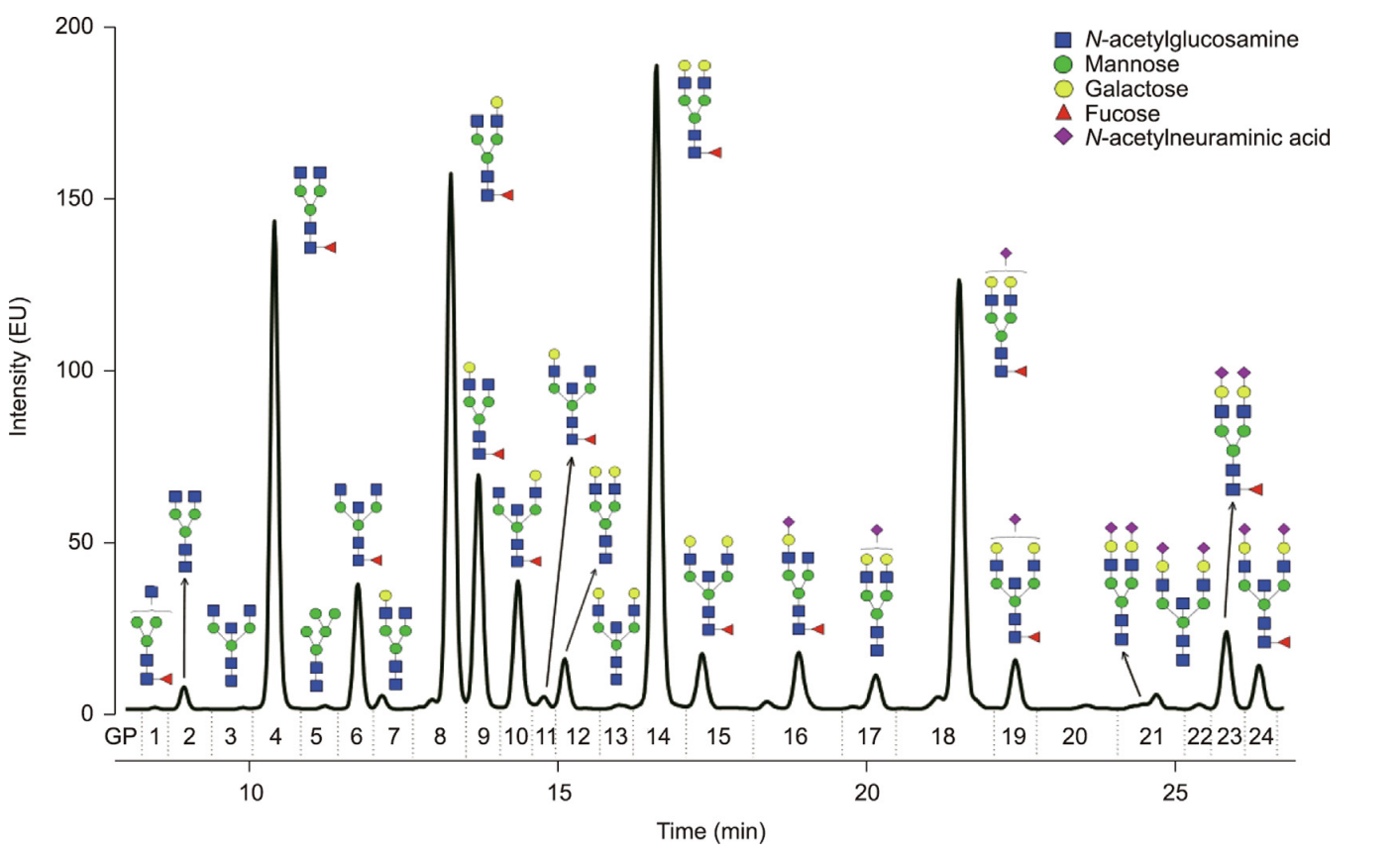


Supplementary Figure 1. List of IgG N-glycan structures corresponding to individual glycan peaks.
